# Supplementary material for: A high-resolution mRNA expression time course of embryonic development in zebrafish
Source: eLife. 2017 Nov 16;6:e30860. doi: 10.7554/eLife.30860 (PMC5690287; doi:10.7554/eLife.30860)
Supplement: Supplementary file 6. [file elife-30860-supp6.zip › biolayout-clusters-files/Cluster253-genes.html]

Cluster253


# Cluster253: Genes

| Ensembl ID | Gene Name | Chr | Start | End | Biotype |
| --- | --- | --- | --- | --- | --- |
| ENSDARG00000078151 | GRIK5 (1 of many) | 19 | 5860832 | 5941840 | protein\_coding |
| ENSDARG00000015003 | arhgap4b | 23 | 18688895 | 18761152 | protein\_coding |
| ENSDARG00000042837 | atp1b3b | 15 | 4362014 | 4424770 | protein\_coding |
| ENSDARG00000006862 | kifap3b | 2 | 23567249 | 23637850 | protein\_coding |
| ENSDARG00000086362 | pou6f2 | 24 | 9898779 | 10131826 | protein\_coding |
| ENSDARG00000087048 | si:dkey-165n16.1 | 16 | 32775193 | 32780735 | protein\_coding |
